# Iliosacral Bone Tumor Resection Using Cannulated Screw-Guided Gigli Saw - A Novel Technique

**DOI:** 10.1186/s12957-021-02349-5

**Published:** 2021-08-16

**Authors:** Tao Ji, Brian Z. J. Chin, Xiaodong Tang, Rongli Yang, Wei Guo

**Affiliations:** 1 Musculoskeletal Tumor Center, People’s Hospital, Peking University, Xizhimen Nan 11#, Xicheng District, Beijing, 100044 China; 2 University Orthopaedics, Hand and Reconstructive Microsurgical Cluster, Singapore, National University Health System, Singapore, Singapore

**Keywords:** iliosacral tumor, osteotomy, surgical margin, limb salvage

## Abstract

**Background:**

Adequate margins are technically difficult to achieve for malignant tumors involving the sacroiliac joint due to limited accessibility and viewing window. In order to address the technical difficulties faced in iliosacral tumor resection, we proposed a technique for precise osteotomy, which involved the use of canulated screws and Gigli saw (CSGS) that facilitated directional control, anteroposterior linkage of resection points and adequate surgical margins. The purpose of the current study was to evaluate whether CSGS technique facilitated sagittal osteotomy at sacral side, and were adequate surgical margins achieved? Also functional and oncological outcomes was determined along with the noteworthy complications.

**Methods:**

From April 2018 to November 2019, we retrospectively reviewed 15 patients who underwent resections for primary tumors of pelvis or sacrum necessitating iliosacral joint removal using the proposed CSGS technique. Chondrosarcoma was the most common diagnosis. The osteotomy site within sacrum was at ipsilateral ventral sacral foramina in 8 cases, midline of sacrum in 5 cases, and contralateral ventral sacral foramina and sacral ala with 1 case each. The average intraoperative blood loss was 3640 mL (range, 1200 and 6000 mL) with a mean operation duration of 7.4 hours (range, 5 to 12 hours). The mean follow-up was 23.0 months (range, 18 and 39 months) for alive patients.

**Results:**

Surgical margins were wide in 12 patients (80%), wide-contaminated in 1 patient (6.7%), and marginal in 2 patients (13.3%). R0 resection was achieved in 12 (80%) patients and R1 resection in 3 patients. There were three local recurrences (20%) occurred at a mean time of 11 months postoperatively. No local recurrence was observed at sacral osteotomy. The overall one-year and three-year survival rate was 86.7% and 72.7% respectively.Complications occurred in three patients.

**Conclusions:**

The current study demonstrated that CSGS technique for tumor resection within the sacrum and pelvis was feasible and can achieve ideal resection accuracies. The use of CSGS was associated with high likelihood of negative margin resections in the current series. Intraoperative use of CSGS appeared to be technically straightforward and allowed achievement of planned surgical margins. It is worthwhile to consider the use of CSGS technique in resection of pelvic tumors with sacral invasion and iliosacral tumors, however further follow-up at mid to long-term is warranted to observe local recurrence rate.

**Supplementary Information:**

The online version contains supplementary material available at 10.1186/s12957-021-02349-5.

## Introduction

Malignant bone tumors surrounding the sacroiliac joint often have poor prognoses due to late diagnoses and significant challenges in surgical managemen t[1–3]. About 25-32% malignant pelvic tumors have sacral infiltratio n[1, 3, 4]. In a bid to decrease rates of local recurrence and achieve wide resection margins, tumors extending to the sacrum were initially managed via hindquarter amputation, at severe cost of patient functionality and quality of lif e[5]. Encouragingly, limb-preserving procedures - primarily in the form of en-bloc tumor resection and reconstruction - emerged as viable surgical alternatives for pelvic sarcomas with sacral infiltration. However, en-bloc resection of iliosacral tumors poses significant challenges due to complex local anatomy, difficulty in nerve root preservations, control of intraoperative bleeding and functional reconstructio n[6, 7]. Resections of pelvic tumors with overly generous margins are generally avoided in limb salvage due to potential anatomic and functional disruptions, thus risking inadequate resection margins and local recurrenc e[1, 2, 8]. Inadequate margin is more often at sacral side due to sacral anatomy and surgical exposur e[9]. Local recurrence was reported to be 21-47% when iliosacral tumors infiltrated the sacru m[1, 10, 11]. As such, tumor resections involving the sacroiliac joint require meticulous preoperative planning, effective and precise osteotomy of affected bone ensuring maximal preservation of neurovascular structures, and holistic postoperative care with regular follow-up.

While multiple reports provide insight on oncological outcomes of malignant tumors spanning the iliosacral joint, there is paucity of technical articles describing effective methods for precise sacral osteotomy - a key component of successful iliosacral tumor resection. Hitherto case reports and case series detailing classifications of sacral tumor locations and their respective positions for sacral osteotomy did not sufficiently portray its technical difficultie s[12, 13], which are often related to limited accessibility and observation. In clinical practice, resection of sacral tumors (of varying locations) demands precise osteotomy through areas such as the sacral ala, medial to the sacral foramina, and the sagittal mid-line of the sacru m[2]. However, techniques involving osteotomes, bone saws, and burrs often prove challenging in the resection of iliosacral tumors via combined antero-posterior approach – in part due to poor linkage between two osteotomy sites, thus risking high intraoperative blood loss and inaccurate resection margins even with adjuvant of intraoperative navigatio n[14]. In the present study, we propose a technique for precise sacral osteotomy in iliosacral resections performed on 15 patients, with early oncological and functional results. This technique involves the use of cannulated screws and Gigli saw (CSGS), which provides benefits of directional control during resection, anteroposterior linkage of resection points, and safe resection margins . The purpose of the current study was to evaluate whether CSGS technique facilitated sagittal osteotomy at sacral side, and were adequate surgical margins achieved? Also functional and oncological outcomes was determined along with the noteworthy complications.

## Materials and Methods

### Clinical Series

From April 2018 to November 2019, patients with iliosacral bone tumors were retrospectively reviewed at a single-tertiary centre. Criteria for inclusion were: (1) histopathologic diagnosis of primary malignant bone tumors involving SIJ, (2) surgical resection necessitating one sagittal osteotomy in sacrum and CSGS technique was used, and (3) follow-up of at least one year for living patients. Additionally, we recorded the locations of tumor epicentre, AJCC tumor staging, and anatomical extent of tumor mass invasion. Institutional review board approval and patient consent were obtained prior to initiation of the study. Demographics of included patients are shown in Table 1. Patients with osteosarcomas and Ewing’s sarcomas underwent neoadjuvant and postoperative adjuvant chemotherapy, whereas those with chondrosarcomas only underwent surgical resection of tumors. MRI after neoadjuvant chemotherapy was performed to determine the soft-tissue margins.

**Table 1:** Demographics of the 15 patients

No	Age/Sex	Primary /Recurrent	Diagnosis*	Location- centric	AJCC Staging	Tumor Extent^¶^	Resection Type♮	Reconstruction**	Estimated Blood Loss (mL)	Operation Time (hours)	Margin	Local Recurrence pattern/ time (m)	Time to metastasis (m)	FU	Status
1	25/F	Primary	OS	Pelvis	T2bN0M0^#^	Sa-PI	Ps IIa	SRS+mesh	2100	6.5	Wide Contaminated/R0	Extensive/10	10	16	DOD
2	32/M	Primary	ES	Sacrum	T2bNxM0	Sm-PI	Ps II/IIIa	SRS+mesh	2800	5	Wide/R0	Localized/17	-	39	NED
3	46/M	Recurrent	CS	Pelvis	T4N0M1a	Sm-PI+II	Ps II	SRS+Autograft	6000	7.5	Wide/R1	-	at Dx	19	DOD
4	47/F	Recurrent	CS	Pelvis	T4N0M0	Scsf-PI	Ps III	SRS+Autograft	5300	7.5	Wide/R0	-	-	36	NED
5	59/M	Primary	OS	Pelvis	T4N0M1b	Ssf-PI+II	Ps IIb	SRS+pelvic prosthesis	6800	12	Marginal/R1	-	3	6	DOD
6	15/F	Recurrent	CS	Pelvis	T4N0M1a	Ssf-PI+II	Ps II	SRS	2500	6	Wide/R0	-	at Dx	11	DOD
7	23/M	Recurrent	OS	Pelvis	T4N0M1a	Ssf-PI+II+III	Ps II	SRS	4000	6	Wide/R0	-	at Dx	33	AWD
8	50/M	Primary	OS	Pelvis	T4N0M1a	Ssf-PI+II+III	Ps IIb	Custom-made pelvic prosthesis	3800	10	Wide/R0	-	-	31	NED
9	28/F	Primary	OS	SIJ^§^	T4N0M0	Ssf-PI	Ps IIa	SRS+Allograft	2500	6	Wide/R0	-	-	27	NED
10	32/F	Primary	CS	Pelvis	T4N0M0^#^	Ssf-PI+II	Ps IIb	Custom-made pelvic prosthesis	5100	11	Wide Contaminated/R1	Localized/9	-	27	AWD
11	29/F	Primary	OS	Pelvis	T4N0M0	Ssf-PI+II	Ps IIb	Custom-made pelvic prosthesis	3500	8	Wide/R0	-	-	21	NED
12	30/F	Recurrent	CS	Sacrum	T3N0M0	Sm-PI	Ps IIa	SRS	1200	5.5	Marginal/R0	-	-	21	NED
13	55/F	Primary	DS	Pelvis	T4N0M0	Sm-PI	Ps IIa	SRS+mesh	2600	7	Wide/R0	-	-	19	NED
14	24/M	Primary	OS	SIJ^§^	T4N0M0	Sm-PI	Ps IIa	SRS+mesh	3900	6.5	Wide/R0	-	-	19	NED
15	24/M	Primary	ES	Pelvis	T4N0M0	Ssf-PI+II	Ps IIb	SRS+pelvic prosthesis	2500	6.5	Wide/R0	-	-	18	NED

* OS, osteosarcoma; ES, Ewing’s sarcoma; CS, Chondrosarcoma; DS, dedifferentiated sarcoma

# Tumor thrombus was found in iliac veins although staged as N0

§The tumor was staged according to both spine and pelvis TNM classification with higher one being recorded.

¶ Sa, sacral ala; Sm, middle line of sacrum, Ssf, ipislateral ventral sacral foramina, Scsf, contralateral ventral sacral foramina

♮According to Peking University Classification ^[reference no.]^

**SRS, screw-rod system

### Surgical Technique

**Fig. 1:**
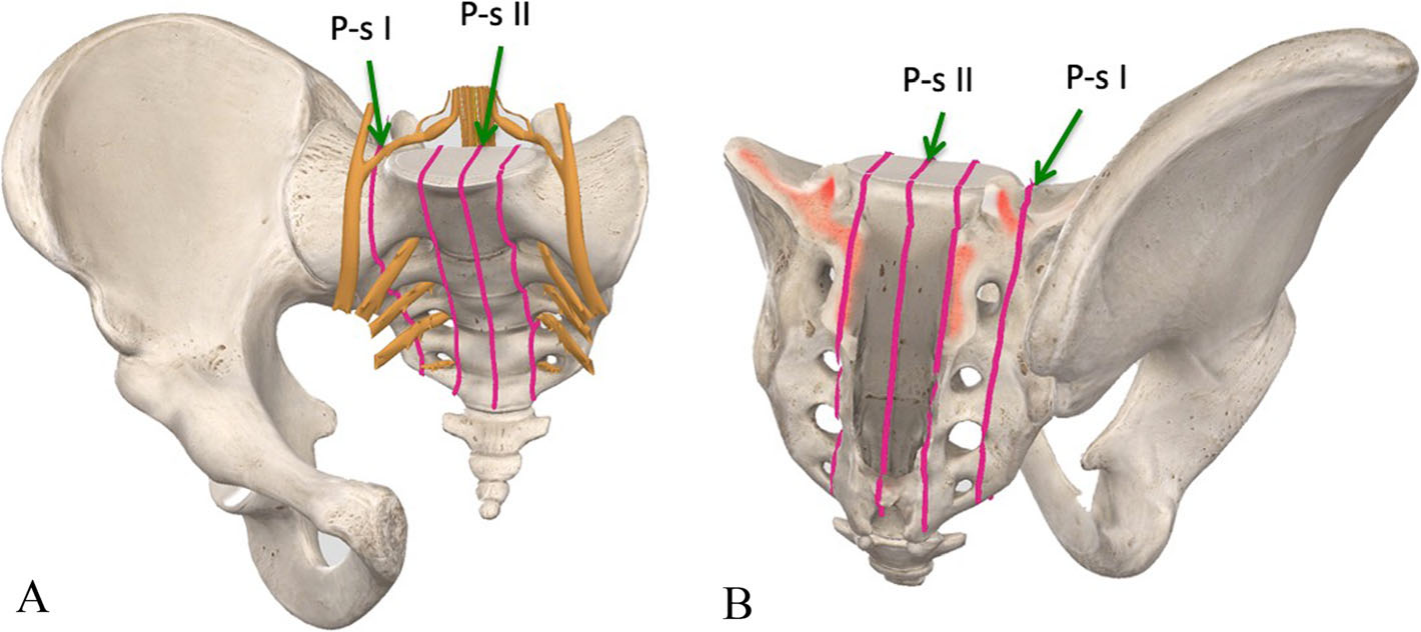
A and B. The typical osteotomy sites at sacral side during iliosacral tumor resection according to the reported classification system [31]. Both anterior view (A) and posterior view (B) were shown

Resections were classified according to the Peking University classificatio n[1] of surgical approaches for pelvic tumors with sacral invasion (Fig. 1): Briefly, pelvisacral (Ps) I, II, and III resections refer to sagittal osteotomies through the ipsilateral wing of the sacrum, through the sacral midline, or lateral to the contralateral sacral foramina, respectively. A Ps a resection describes a pelvic osteotomy through the ilium, whereas a Ps b resection describes a concurrent resection of the acetabulum with osteotomies performed through the pubis and ischium or the pubic symphysis

**Fig. 2:**
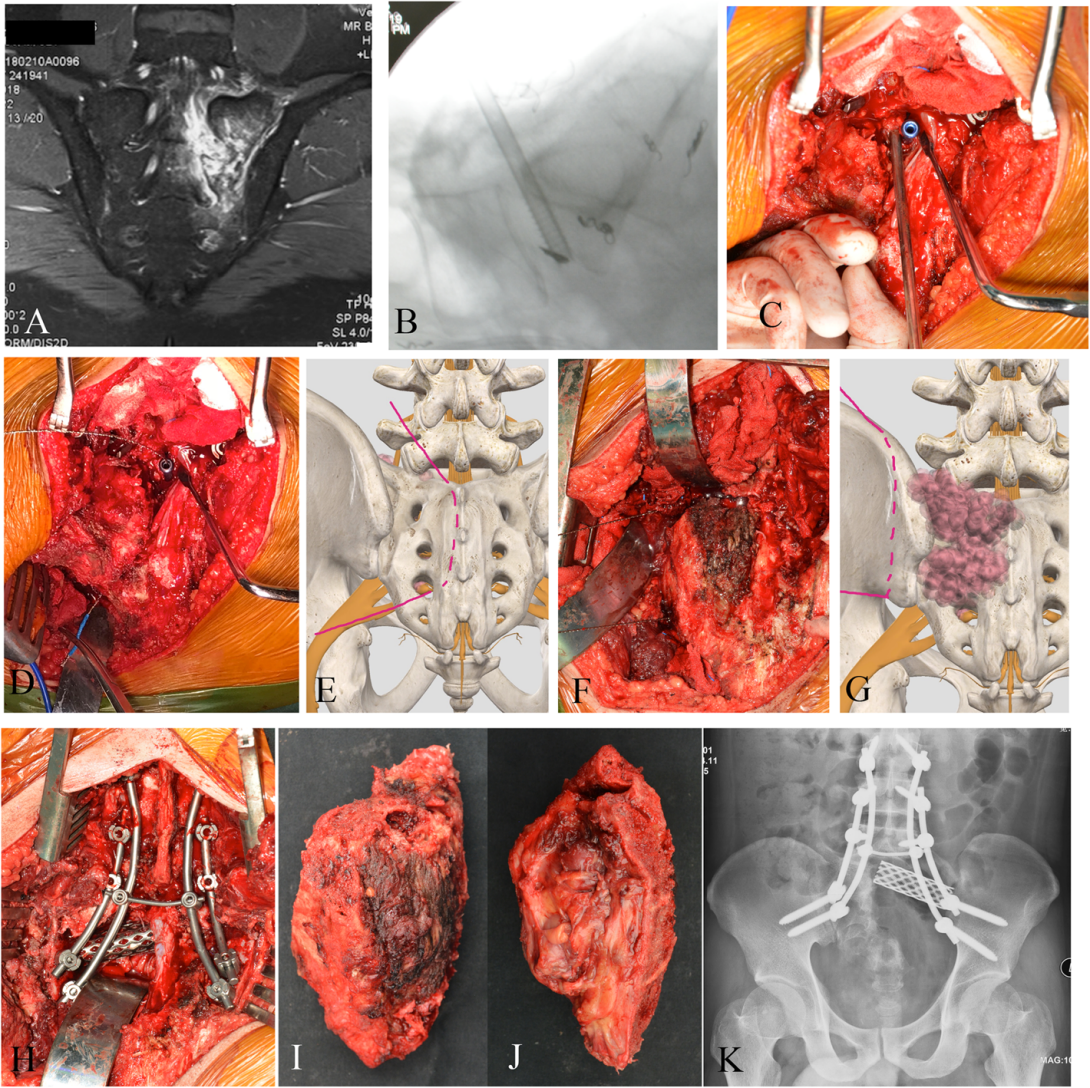
A-K. An illustrative case (patient no. 2) of CSGS technique. Preoperative MRI after neo-adjuvant chemotherapy showed tumor extent (A). A canulated screw was placed at middle point of L5/S1 level without penetration of the anterior cortex under fluoroscopic guidance (B). Penetration of the cannulated screw through the anterior cortex was confirmed by finger palpation by a pedicle sound (C). D and E showed a Gigli saw was introduced through the canulated screw and the planned osteotomy site (dash line on figure E). Then the iliac osteotomy was carried out through posterior approach (F and G). The tumor was removed after resection of ipsilateral half of L5/S1 disk (H). The specimen was showed (I and J). Postoperative X-ray showed osteotomy sites and reconstruction

**Fig. 3:**
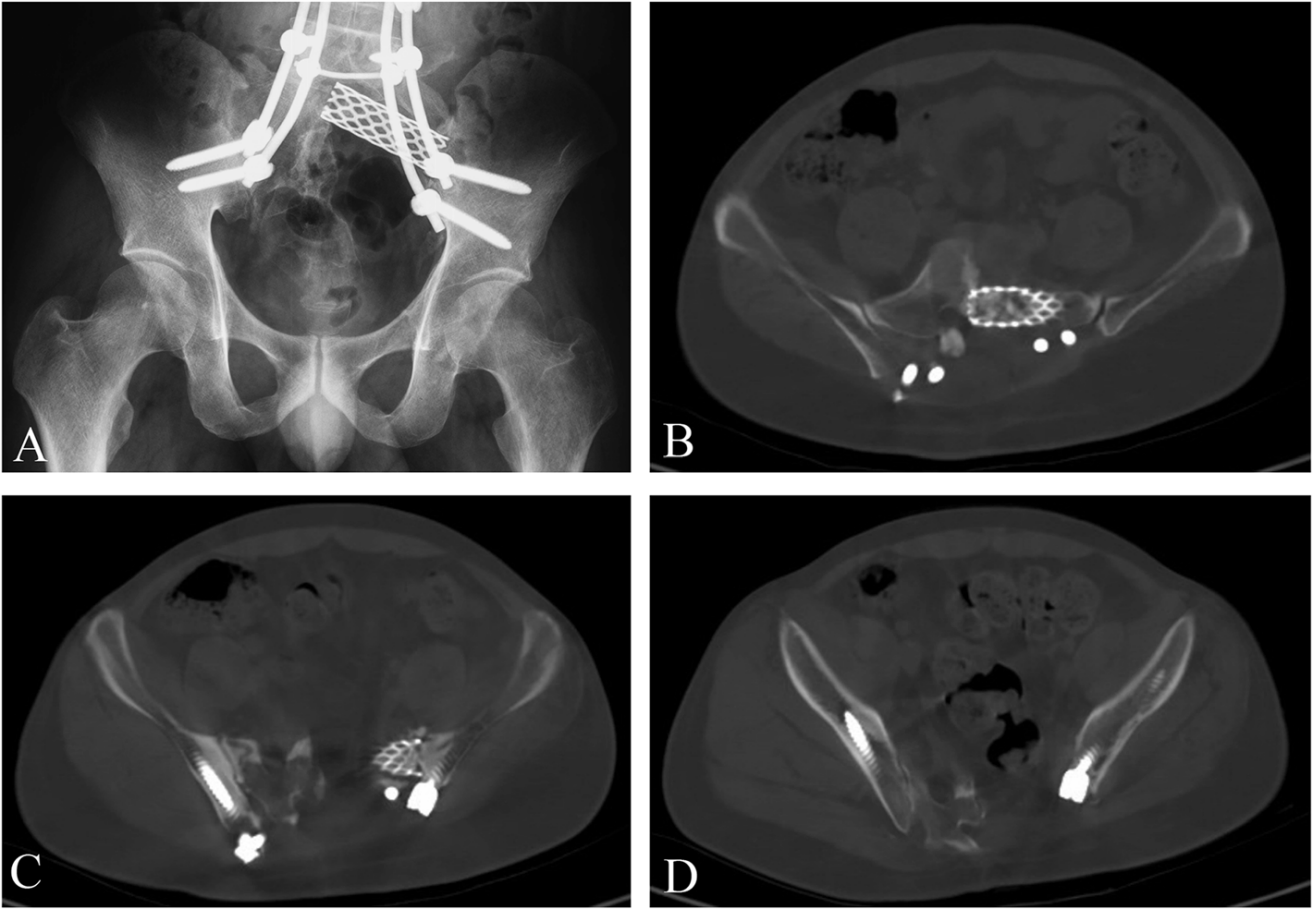
A-D. The follow-up at one and half year of patient no. 2. The patient can walk independently with mild gait abnormal. Pelvic x-ray were shown. The transverse CT scan (B, C and D) of different levels showed bony cut at both sacrum and ilium

**Fig. 4:**
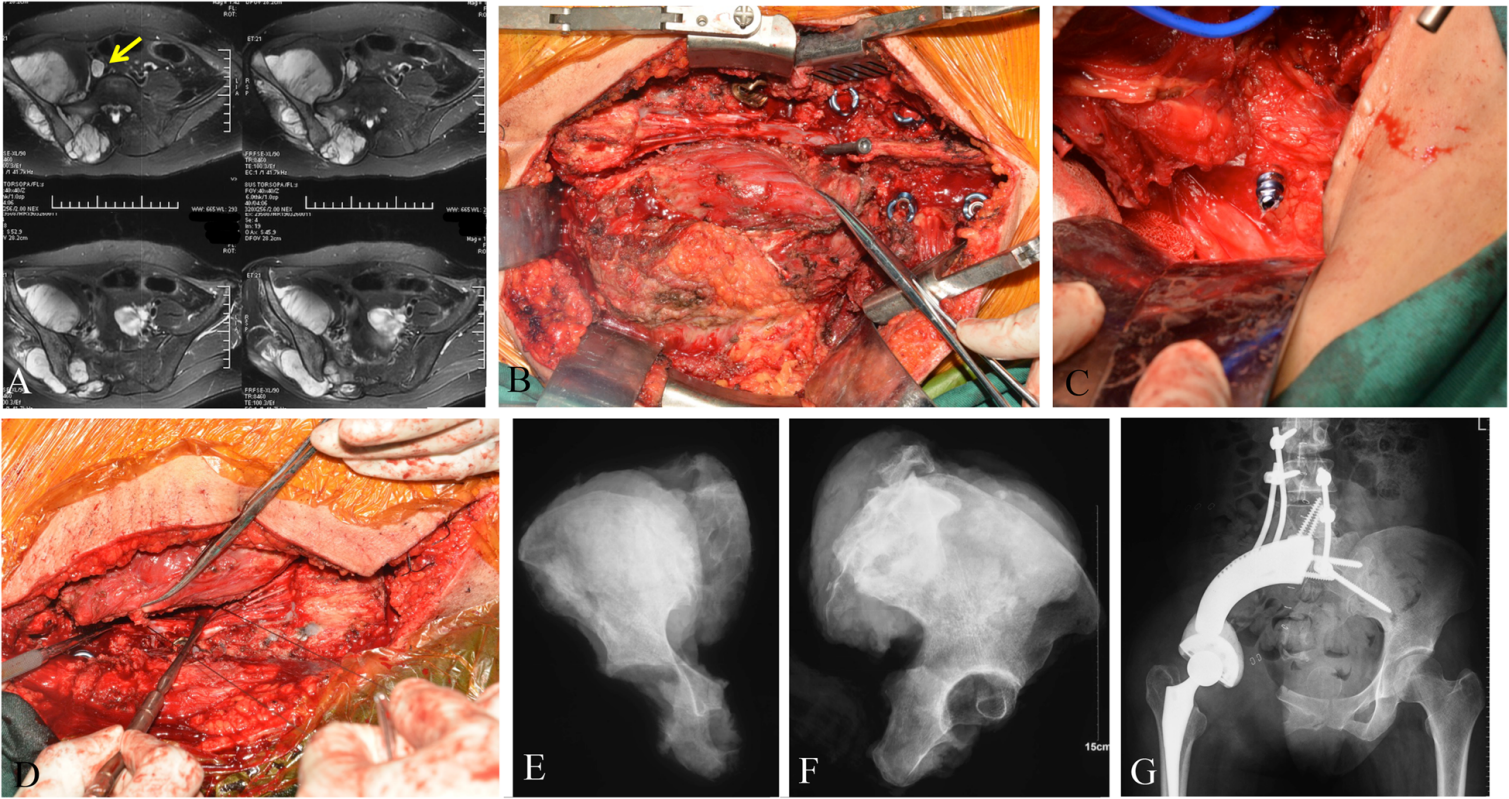
A-G. The number 10 patient was diagnosed of chondrosarcoma in right pelvis invading sacrum. Preoperative MRI (A) showed tumor thrombus in iliac vein (arrow). Following sacral laminectomy, affected nerve roots (S1-3) were identified and ligated during posterior approach. Then the canulated screw as placed (B). Intraoperatvie photo of anterior approach (C) showed the canulated screw advanced through the anterior sacrum under direct observation. Then the Gigli saw was placed and sagittal osteotomy at sacrum was performed (D). X-rays (E and F) of the specimen were shown. A custom-made pelvic endoprosthesis combined with SRS were used to reconstruct the defect (G)

**Fig. 5:**
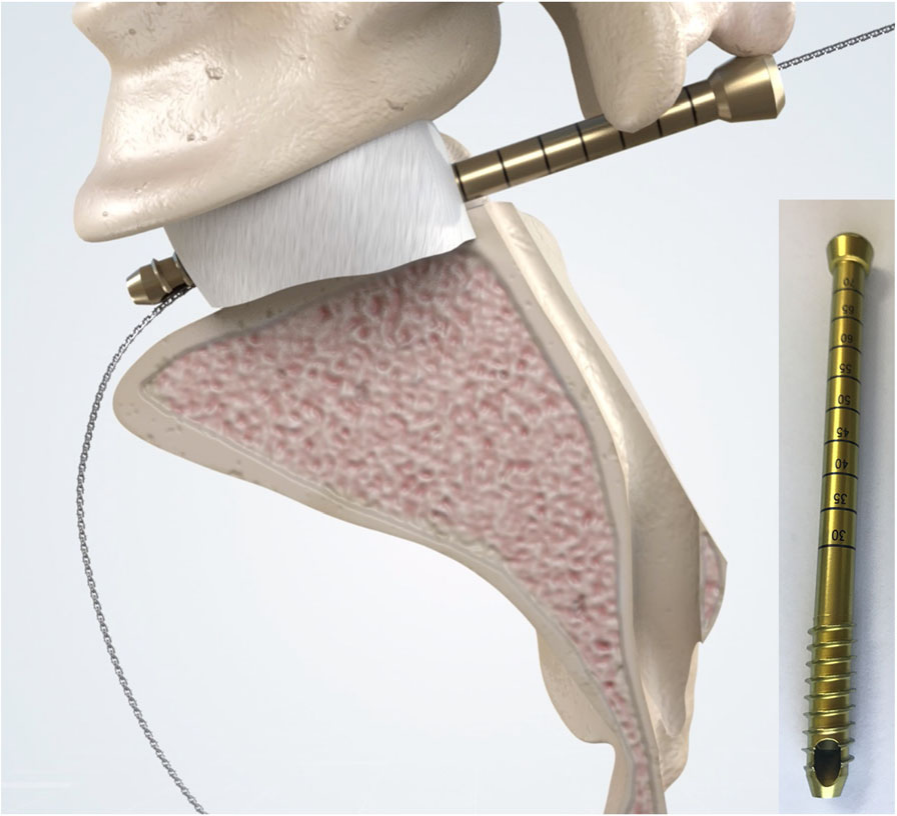
A modified canulated screw was developed with purpose of direction-control of the Gigli saw. This can be helpful for posterior approach only procedures

All procedures were performed begin with the patient in a prone position (posterior approach): A midline posterior reverse Y-shape incision was first performed, followed by L4/5 pedicle screws and contralateral S2-alar-iliac screw fixation. Exposure of the posterior cortex of sacrum and sacroiliac joint was achieved via gluteaul flap dissection. To the greatest extent, exposure of posterior 1/3rd of the ilium can be achieved (sagittally guided by the greater sciatic notch). If the tumor margins do not exceed the aforementioned anatomical landmark, a Gigli saw can be placed at the planned iliac osteotomy site with confirmation of safety margins around the tumor (Figs. 2 and 3). Otherwise, an anterior approach would be needed to expose and dissect structures anterior to the acetabulum – up to the symphysis pubis (Fig. 4). Sacral laminectomy was performed for identification and ligation of affected nerve roots (S1, S2) for all included patients due to sagittal invasion of tumor to the ipsilateral sacral foramina. If the ipsilateral side S3 and below nerve can be preserved, piezoelectric osteotomy can be performed from middle of S3/4 to lower edge of sacroiliac joint to mobilize the S3 nerve root. The bottom of sacral canal at L5/S1 level can be exposed with dura retractor. Next, a pedicle probe was used to locate the sacral midline at L5/S1 level for resection of type P-s II, with subsequent insertion of cannulated screw through (without penetration of the anterior cortex) under fluoroscopic guidance (Fig. 2C). Penetration of the cannulated screw through the anterior cortex was confirmed by finger palpation by a pedicle sound. According preoperative plan, the canulated screw can be placed at lateral recess for resection medial to the ipsilateral sacral foramina (between type P-s I and P-s II resection). Thereafter, a Gigli saw was introduced through the cannulated screw, allowing for sagittal osteotomy of S1-3 to be performed on the ipsilateral side (thus preserving the ipsilateral S3-5 nerves and bony structures of S4-5 with coccyx) or the sagittal osteotomy of S1-5 depending on the extent of tumor invasion as shown in Supplemental Digital Content. Then, iliac osteotomy was carried out as previously reported for total sacrectomy through single posterior approach onl y[15]. Ipsilateral half of L5/S1 disk removal can be performed with specimen being mobilized and distracted, thus achieving a standard SIJ (type IV) resection by posterior approach only. A modified canulated screw was developed as well with purpose of direction-control of Gigli saw (Fig. 5) An animation of said CSGS technique for sacral osteotomy is available as supplementary material.

For patients with sacral tumor extending anteriorly beyond the posterior 1/3rd of the iliac bone, a combined anterior and posterior approaches were necessary. The canulated screw was usually placed at the L5/S1 level and the posterior wound was then closed temporarily prior to commencement of the anterior approach, performed with the patient lying in the lateral position. During the anterior approach, the tumor was exposed via soft tissue dissection, along with dissection of the iliacus and gluteus muscles. Commonly, iliac vessels and the lumbosacral trunk are dissected and protected. The anterior aspect of the sacrum promontary was then exposed. For patients with tumor involvement of the ilium without acetabular involvement (Ps-a), a distal cut was made via supraacetabular osteotomy. Patients with acetabular involvement (Ps-b) underwent osteotomies at the ischium and pubis, or through the pubic symphysis. During this step, the wound of posterior approach was then reopened. The canulated screw was then slowly advanced through the anterior sacrum under direct observation (Fig. 4-C). Once through, a Gigli saw was then introduced through the cannulated screw in preparation for osteotomy. Osteotomy was performed once confirmation of osteotomy site and protection of dura and nerve roots were completed (Fig. 4-D). All resected sections were oriented, landmarked, then sent for surgical margin evaluation by an experienced histopathologist. Reconstruction was achieved using titanium mesh cage and pedicle screw-rod system across all patients with intact acetabulum, whereas patients with acetabular resection underwent fixation comprising of interpedicular screw fixation combined with hemipelvis reconstruction, extending up to the L3 vertebra e[16].

### Postoperative course

At 4 weeks postoperatively, patients were allowed to touch-toe weight bear, with gradually increased weight-bearing from 6 weeks postoperatively onwards. Hip flexion beyond 90° was only allowed 6 weeks postoperatively for patients with acetabulum reconstruction. Postoperative follow-up of oncological outcomes, complications and function were conducted at regular intervals of 3 months for a minimum of 2 years. Resection margins were categorized by both Enneking syste m[17], wide, wide-contaminated, or marginal; and TNM R syste m[18]. Evaluation of functional outcomes was performed using the Musculoskeletal Tumor Society (MSTS) rating scale, and the MUD scoring system devised by Huang et a l[19] for comprehensive evaluation of neurological function following sacrectomy. Briefly, MUD scoring system involves three main domains (motor function & sensation of lower limbs (M), urination & uriesthesia (U), defecation & rectal sensation (D)) comprising scores from 0 to 3 for each domain, with higher scores indicating a higher level of function for both measures.

### Statistical Analysis

SPSS v. 19 (IBM, Armonk, New York, USA) was used for analysis. Continuous variables were compared using independent-samples t-tests. Local recurrence-free, disease-free, and overall survival were calculated to estimate the survival with Kaplan-Meier survival analysis. A p-value < 0.05 was considered statistically significant.

## Results

A total of 15 patients (53.3% female) with a mean age of 34.6 years (15-59) were enrolled in this study (Table 1). Chondrosarcoma (n = 7, 46.7%) was the most common diagnosis, with other diagnoses including: osteosarcoma (n = 5, 33.3%), Ewing’s sarcoma (n = 2, 13.3%) and dedifferentiated sarcoma (n = 1, 6.7%). According to the Peking University classification of surgical approaches for pelvic tumors with sacral invasion, 4 patients underwent pelvisacral (Ps) II resections, 5 underwent Ps IIa resections, 5 underwent Ps IIb resections, and 1 underwent a Ps III resection (Table 2). Reconstruction techniques ranged from screw-rod system (SRS) reconstruction with mesh or tibial autograft for Ps II cases, to SRS reconstruction with custom-made pelvic prosthesis for Ps IIb cases. The average intraoperative blood loss was 3640 mL (1200–6000 mL), with a mean operative duration of 7.4 hours (5-12 hrs) documented in surgical notes.

**Table 2:** Functional outcome and complications

No	Age/Sex	Procedure	MSTS 93 (%)	MUD Score (%)	Complications	
1	25/F	LS	43.3	48.1		
2	32/M	LS	76.7	70.4	Hardware breakage	
3	46/M	Amputation	-	-		
4	47/F	Amputation	-	-		
5	59/M	LS	23.3	33.3	Wound dehiscence	Debridement + Flap
6	15/F	Amputation	-	-		
7	23/M	Amputation	-	-		
8	50/M	LS	36.7	70.4		
9	28/F	LS	53.3	81.4		
10	32/F	LS	26.6	51.9		
11	29/F	LS	33.3	74.1	Wound dehiscence	Debridement
12	30/F	LS	60.0	74.1		
13	55/F	LS	50	40.7		
14	24/M	LS	56.7	77.8		
15	24/M	LS	36.7	70.4		

### Oncological Outcomes

Most patients presented with localized disease (n = 10, 66.7%), whereas 5 patients (33.3%) had metastases at time of diagnosis - three of which were found to have pulmonary metastases. Surgical resection margins were wide in 12 patients (80%), wide-contaminated in 1 patient (6.7%), and marginal in 2 patients (13.3%). R0 resection was achieved in 12 (80%) patients and R1 resection in the other three patients. In patient no. 3, the R1 margin was found at sacral osteotomy. The R0 resection was achieved at sacral side in all the other patients.

**Fig. 6:**
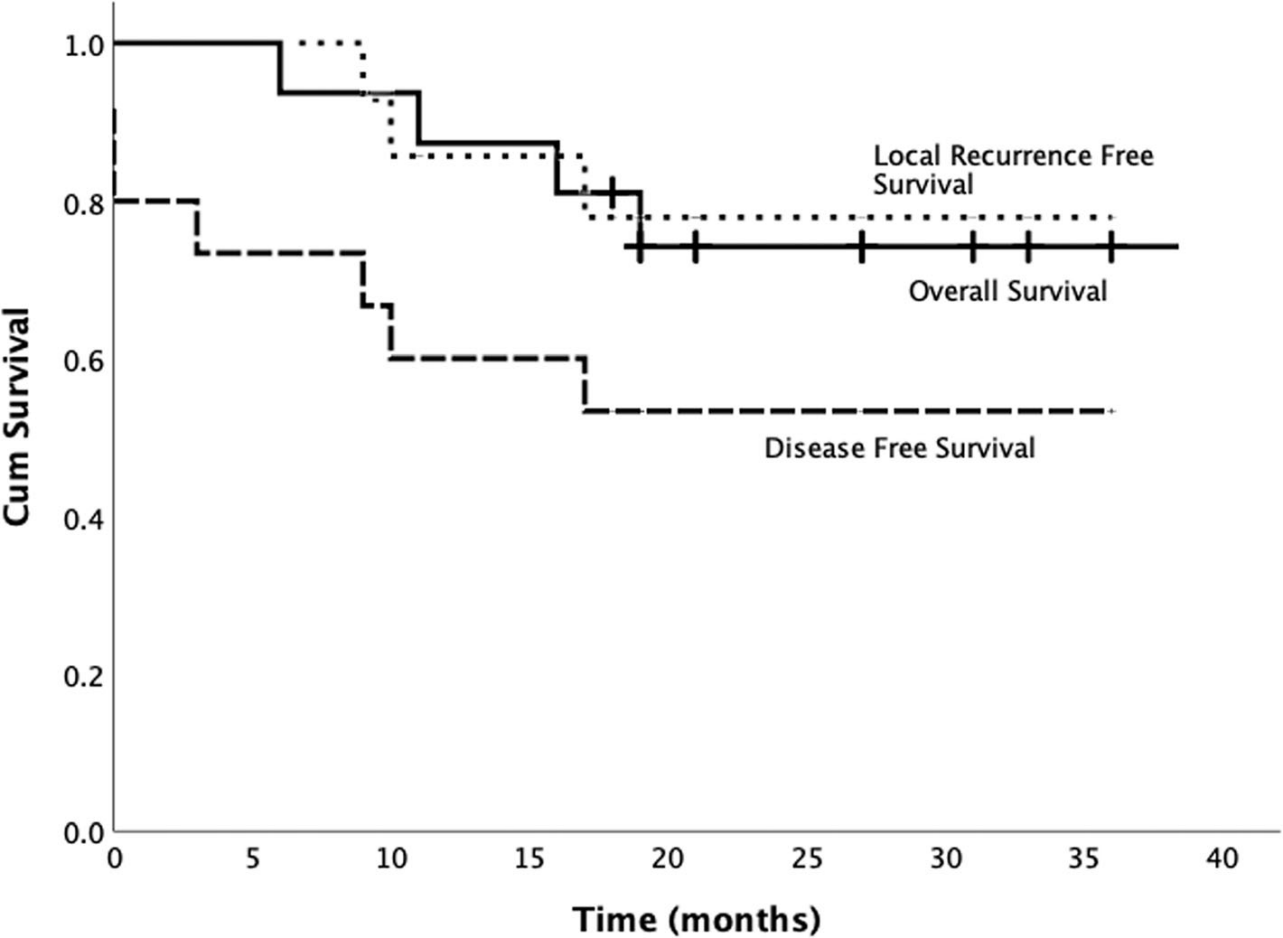
The Kaplan-Meier survival curves shows overall survival, local-recurrence-free survival and disease-free survival

The overall one-year and three-year survival rate was 86.7% (95% CI: 69.5; 103.9 ) and 72.7% (95%CI: 49.8; 95.6). Local recurrence free survival was 85.7% (95% CI: 67.3; 104.1) and 77.9% (95% CI: 55.8; 100.0) respectively at one year and three years. One-year and 3-year disease-free survival was 60% (95% CI: 35.3; 84.7) and 53.3% (95% CI: 28.0, 78.6) respectively (Fig. 6).

At mean follow-up of 23.0 months (18-39), nine patients (60%) were alive with no evidence of disease, one (6.7%) was alive with pulmonary metastasis, one (6.7%) was alive with local recurrence, and four patients (26.6%) had died of disease. There were three local recurrences (20%) occurred at a mean time of 11 months after surgery, although R0 resection was achieved in two of the three patients. All three patients received limb-salvage procedures. No local recurrences were found among the four patients who underwent amputations. No localized recurrence was observed at the sacral osteotomy sites. Patient No.1 diagnosed with chondroblastic osteosarcoma developed extensive tumor recurrence at 10 months postoperatively soon after adjuvant chemotherapy, and eventually died of disease at 16 months due to rapid systemic progression. In patient No.2, a 1.5cm soft tissue local recurrence adjacent to bladder was found at a routine follow-up 17 months after surgery. He received stereotactic radiotherapy and remained alive without disease at most recent follow-up. Patient no.10 was found to have local recurrence at 9 months postoperatively with tumor invasion of the iliac lymph nodes and pubic bone, and extension of tumor thrombus into the iliac vein and inferior vena cava despite prior removal of tumor thrombus in iliac vein during primary surgery. Targeted therapy was started and disease was stable till most recent follow-up.

### Functional Outcomes

Functional outcomes were collected for all patients at a minimum of 12 months follow-up, with no patients lost to follow-up (Table 2). The mean MSTS-93 score across all 11 patients (73.3%) who underwent limb salvage surgery was 45.1% (SD 16.0, 23.3 to 76.7), whereas the mean MUD score was 63.0% (SD 16.4, 33.3 to 81.4). Patients who underwent resections involving the acetabular component had a lower mean MSTS-93 score ( 31.3, 23.3 to 36.7) than patients who underwent resections with sparing of the acetabulum (56.7, 43.3 to 76.7) (p=0.02).

### Complications

A total of 3 patients (20%) had postoperative complications (Table 2). One patient suffered from wound dehiscence, which was managed with a combination of intravenous antibiotics, operative washout, and wound debridement with subsequent wound closure. Another patient with wound dehiscence was also managed similarly, with additional rotational flap performed for adequate closure. The latter patient received postoperative radiotherapy at 60Gy due to inadequate margins at periacetabular region. Both patients underwent wound closures only upon confirmation of negative bacterial and wound cultures. One rod breakage of SRS was observed in one patient with reconstruction of SRS and mesh.

## Discussion

Despite advancements in surgical techniques and medical technology, precise surgical resection of iliosacral bone tumors still prove daunting even for experienced orthopaedic surgeons. In our previous experiences, sacral osteotomies using osteotome, burrs and piezoelectric osteotomy prove challenging due to limited accessibility into the pelvisacral space and poor directional control. This often resulted in unsatisfactory resection margins with excessive damage to neighbouring bone and anatomical structures – with the sequelae of increased local recurrences and greater patient morbidity. To circumvent these recurring issues, a novel technique for osteotomy of sacral tumors was trialled using cannulated screws and Gigli saws for iliosacral tumor resections. The proposed technique was mainly based on the principle of a man-made bony canal that can accommodate a Gigli saw. The placement of canulated screw can be guided by intraoperative navigation or PSI. The advantage of Gigli saw was completeness, which was extremely helpful at sacral resection. It was useful particular when sagittal hemisacrectomy was required. Intraoperatively, placement of the cannulated screw through the sacrum provided adequate access into the pelvisacral space, with subsequent resection using Gigli saw granting directional control as we navigated around tumor margins. The effectiveness of this technique was assessed via analyses of surgical margins, oncological, and functional outcomes of 15 patients with varying extents of pelvisacral resection – ranging from Ps IIa (tumor involvement of ipsilateral sacral foramina with sparing of the acetabulum) to Ps IIIb (tumor involvement lateral to contralateral sacral foramina with involvement of acetabulum) and osteotomy between Ps-II and Ps-III. The classification system requiring more accurate in executing osteotomy.

In our patient cohort, chondrosarcoma was found to be the predominant pathology (46.7%), a consistent finding with multiple publication s[2, 6, 7, 20, 21]. Despite complexity of iliosacral sarcoma resections, satisfactory surgical margins were achieved with a 80% negative margin (R0) rate, compared with that reported in literature for iliosacral resections (Table 3). The authors attribute the above to the use of cannulated screws and Gigli saw, which allows for better linkage of anterior and posterior aspects of the sacrum, easy access into the pelvisacral space and precise direction of osteotomy within the affected sacrum. Intraoperative navigation or PSI has gradually been accommodated in complex osteotomy in pelvic tumor surgery. The positive margin was reported to be 11.1- 18.2% with use of navigation or PSI, which was lower than that of 25.0-34.4% by free-hand [7, 14, 22, 23]. Our results showed a positive margin of 20% lower than those not using intraoperative adjuvant techniques, which can be attributed to the use of CSGS technique. However, the positive margin rate may potentially be further decreased if the proposed CSGS technique are used in conjunction with computer-assisted techniques, thereby improving both identification of planned osteotomy site and execution of bony cutting. The overall local recurrence in the current study was 20% among the range of literature reported recentl y[1, 14, 21]. One patient (no. 10) with contaminated wide margins suffered from local recurrence at 6 months follow-up. The recurrent tumor was found at the pubic osteotomy site, concomitant with an iliac tumor thrombus. A tumor thrombectomy was performed concurrent with resection of the primary tumor. Patient no.1 with wide resection margin experienced extensive tumor recurrence at 10 months follow-up. For this patient, the authors believe the cause of extensive tumor recurrence (despite wide resection margins) to be aggressive clinical behaviour of chondroblastic osteosarcoma, resulting in widespread extension of tumor within the entire primary operation field resulting in rapid systemic progression.

**Table 3:** Comparative studies of surgical treatment for iliosacral or pelvic tumors

Study	Year	No. of Patients	Assisted Technique*	Mean Followup (month)	Positive Margins (%)	Local Recurrence% (month)	Complication Rate (%)
Gupta et a l[7]	2020	32	SA	159	34.4	3 (26)	53.1
Evrard et a l[22]	2019	28	9 PSI, 19 SA	52	PSI 11.1, SA52.6	PSI 0, SA 36.8 (<12)	10.5
Laitinen et a l[24]	2017	35	SA	110	29.7	42.2 (24)	18.8
Laitinen et a l[14]	2017	21	9 IN, 12 SA	44.6	IN 11.1, SA 25.0	IN 22.2, SA 50.0	33.3
Gouin et a l[23]	2014	11	PSI	13	18.2	9.1 (18)	72.7
Current study	2021	15	SA	22.9	20	20 (12)	20

* PSI, patient-specific instruments; SA, surgery alone; IN, intraoperative navigation

Functional outcomes for our patients who underwent limb salvage procedures are promising despite significant morbidity associated with iliosacral resections and subsequent reconstruction. In our series, the mean MSTS93 score of 45.1% is comparable with other studies in literatur e[1, 6, 21, 24], whereas the mean MUD score is 63%. Patients who underwent acetabular resection were found to have lower mean MSTS93 and MUD scores compared to patients with acetabular-sparing procedures. This can be attributed to significant gait abnormality due to loss of hip abductor muscles from resection of the ilium. Additionally, patients also suffer from partial loss of lower limb function due to sacrifice of sacral nerve roots from the lumbosacral trunk. In terms of bowel and bladder function, sacrifice of unilateral sacral nerves resulted in loss of sensation on the ipsilateral side, akin to results seen in sagittal sacrectomy as previously reporte d[19, 25].

Complication rates following iliosacral tumor resections vary in literature. Of note, existing literature on iliosacral resections with reconstruction often report complication rates of 50% or mor e[1, 6, 7]. In this case series, wound complications were by far the most common complication, with two patients (13.3%) developing wound dehiscence requiring intravenous antibiotics, further surgical debridements and wound closure. Both patients underwent successful washouts and adequate wound healing with no further complications at latest follow-up. Encouragingly, despite lengthy operative durations and large resections (involving Ps IIa to Ps IIIb resections), there were no reported major neurovascular, soft tissue or reconstructive complications (e.g. aseptic loosening, implant infection) in our cohort at latest follow-up. While this is in part due to significant surgical experience in a high-volume tumor centr e[1, 16, 26], the proposed technique for iliosacral resection also mitigated potential damage to neurovascular structures and viscera, and creation of satisfactory defects which allowed for ease of reconstruction.

This study is not without limitations. The retrospective nature of our study, with lack of a control group, limits recruitment of large number of patients. However, the authors believe this to be due to the novelty of the proposed technique, with patient numbers sufficient to show results. Additionally, mean follow-up time is short for this case series, where longer-term follow-up may have shown increase in local tumor recurrences, reflected more chronic complications such as aseptic loosening, and demonstrate change in functional/oncological outcomes with time. The present study focused mainly on the description of the technique, however the authors are continuing to follow-up with patients from the present case series, with aims to report medium-term and long-term outcomes of our technique. Lastly, the authors believe this novel technique of iliosacral tumor resection could be augmented further with use of navigation and more precise preoperative planning. As our centre does not have navigational systems at present, we believe that the combination of both may produce promising results, allowing for greater resection precision with potentially lower rates of local recurrence and better functional/oncological outcomes.

## Conclusion

The current study demonstrated that CSGS technique for tumor resection within the sacrum and pelvis was feasible and can provide satisfying cutting accuracy. The use of CSGS was associated with high likelihood of negative margin resections. Intraoperative use of CSGS appeared to be easy-to-handle and allowed achieving planned surgical margins. It is worthwhile to consider the use of CSGS technique in resection of pelvic tumors with sacral invasion and iliosacral tumors, however further followup is warranted.

## Supplementary Information

**Additional file 1.**

## Data Availability

The datasets used and/or analyzed during the current study are available from the corresponding author on reasonable request.
